# Therapeutic implications of quercetin and its derived-products in COVID-19 protection and prophylactic

**DOI:** 10.1016/j.heliyon.2024.e30080

**Published:** 2024-04-30

**Authors:** Wan-Yi Ho, Zi-han Shen, Yijing Chen, Ting-Hsu Chen, XiaoLin Lu, Yaw-Syan Fu

**Affiliations:** a Department of Anatomy, Kaohsiung Medical University, Kaohsiung, Taiwan; b Department of Clinical Medicine, Xiamen Medical College, Xiamen, 361023, Fujian, China; c Department of Dentisty, Xiamen Medical College, Xiamen, 361023, Fujian, China; d Graduate Institute of Brain and Mind Sciences, College of Medicine, National Taiwan University, Taipei, 10051, Taiwan; e Institute of Respiratory Disease, Department of Basic Medical Science, Xiamen Medical College, Xiamen, 361023, Fujian, China; f Anatomy Section, Department of Basic Medical Science, Xiamen Medical College, Xiamen, 361023, Fujian, China

**Keywords:** Quercetin, COVID-19, *In silico*, Long-COVID, MIS

## Abstract

Severe acute respiratory syndrome coronavirus 2 (SARS-CoV-2), a novel human coronavirus, which has triggered a global pandemic of the coronavirus infectious disease 2019 (COVID-19). Outbreaks of emerging infectious diseases continue to challenge human health worldwide. The virus conquers human cells through the angiotensin-converting enzyme 2 receptor-driven pathway by mostly targeting the human respiratory tract. Quercetin is a natural flavonoid widely represented in the plant kingdom. Cumulative evidence has demonstrated that quercetin and its derivatives have various pharmacological properties including anti-cancer, anti-hypertension, anti-hyperlipidemia, anti-hyperglycemia, anti-microbial, antiviral, neuroprotective, and cardio-protective effects, because it is a potential treatment for severe inflammation and acute respiratory distress syndrome. Furthermore, it is the main life-threatening condition in patients with COVID-19. This article provides a comprehensive review of the primary literature on the predictable effectiveness of quercetin and its derivatives docked to multi-target of SARS-CoV-2 and host cells via *in silico* and some of validation through *in vitro*, *in vivo*, and clinically to fight SARS-CoV-2 infections, contribute to the reduction of inflammation, which suggests the preventive and therapeutic latency of quercetin and its derived-products against COVID-19 pandemic, multisystem inflammatory syndromes (MIS), and long-COVID.

## Graphical abstract

**Figure:**
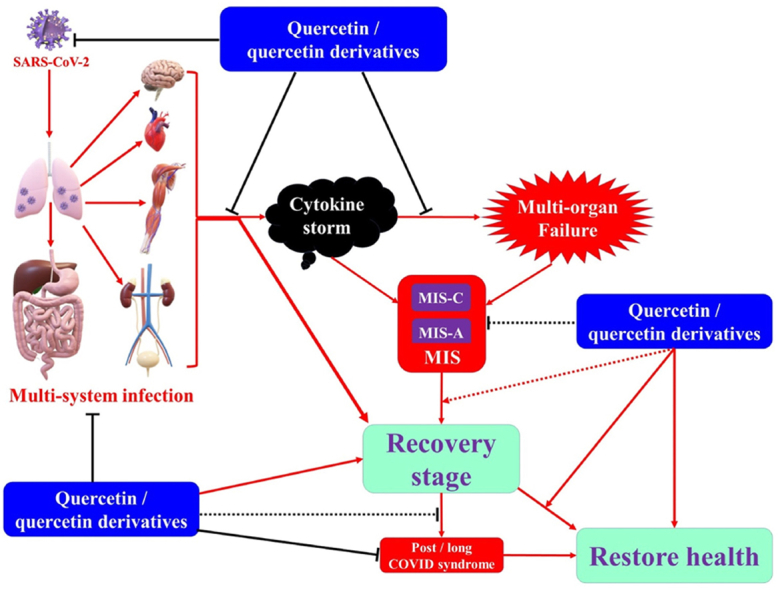

## 1 Introduction

The first case of severe acute respiratory syndrome coronavirus 2 (SARS-CoV-2) was reported clinically and identified in Wuhan City, China, in December 2019. This was followed by a rapidly growing outbreak and transmission worldwide, which caused the most serious epidemic in human history (https://covid19.who.int/). At the end of 2019, the disease associated with SARS-CoV-2 was officially named coronavirus disease 2019 (COVID-19) by the World Health Organization (WHO) [1]. Based on data recorded by the WHO regarding the COVID-19 pandemic that has spread to all countries and regions, over 774 million people were infected and more than 7.03 million deaths occurred before Feb 2024 (https://covid19.who.int/), with both large between- and within-country variations in COVID-19 death rates. Infections result in a broad spectrum of clinical disease severity, which has led to devastating health crises worldwide [2]. First, SARS-CoV2 binds to a serine protease, which acts as a receptor for the ectoenzyme angiotensin-converting enzyme 2 (ACE2), whereas another transmembrane serine protease 2 (TMPRSS2) is necessary for priming the viral spike protein required to enter the cell, leading to COVID-19 [3,4]. This severity originates from the immune response of the host, particularly the release of inflammatory cytokines. Some of the consequences of severe inflammation and cytokine storms include acute lung injury, acute respiratory distress syndrome (ARDS), and multiple organ dysfunction syndromes.

Currently, no treatment exists to counter this highly contagious disease with high viral variants; some vaccines (AstraZeneca AZ, BioNTech BNT, Moderna) and antiviral agents (Remdesivir, Paxlovid (Pfizer), or Molnupiravir (Merck)) have been used for emergency authorization use (EAU), as stipulated by the WHO or Food and Drug Administration (FDA). At present, the WHO and all countries have announced the end of the emergency phase of the COVID-19 pandemic for more than one year, but the new mutant variants of SARS-CoV-2 continue to spread and cause reinfections in several million people [5], and the overall reinfection rate in Asian populations is more than 9.5 % per year [6]. Thus, there is an urgent need to find a cure for the disease, and global efforts have been directed toward developing SARS-CoV-2-specific antivirals and immunomodulators. Notably, potential and effective phytochemicals (phytoconstituents, phytomolecules, and phytocompounds), which act as phytomedicines, have been investigated to meet global demands [7]. In this review, we focus on recent investigations of the interactions of quercetin (or quercetin derivatives) with hosts or viral targets that interfere with the viral infective cycle of SARS-CoV-2. We collate and organize the most published results of the effects quercetin and quercetin derivatives on SARS-CoV-2 based on in-silico simulations, in-vitro and in-vivo tests, and clinical trials to reveal the therapeutic value of these molecules. Such promising studies may stimulate the use of quercetin, quercetin derivatives, and other phytocompounds in preventive/cotherapeutic strategies to combat the COVID-19 pandemic.

## 2 The viral structures and proteins of SARS-Co-2

SARS-CoV-2 is a single positive-stranded ribonucleic acid (RNA) virus, whose mutation rate is higher than that of DNA viruses [8]. To date, recorded variants of SARS-CoV-2 are over 1000, including the major epidemic variants, Beta, Delta, and Omicron [9] (https://www.who.int/activities/tracking-SARS-CoV-2-variants/tracking-SARS-CoV-2-variants). The diameter of SARS-CoV-2 viral particles is approximately 100 nm, and SARS-CoV-2 is enclosed by a phospholipid bilayer envelope with three integral membrane proteins: spike (S), envelope (E), and membrane (M) proteins [10] (Fig. 1). Within the envelope of the SARS-CoV-2 viral particles, there are single positive-stranded viral RNA genomes with a length of approximately 30 kb; these genomes bind to nucleocapsid (N) proteins to form tight spiral-folding structures, such as helical ribonucleoproteins (RNPs) [1,11]. Stable interactions between RNPs and M-proteins are involved in strengthening the coronavirus assemblage and may protect the virus against environmental and physical challenges [11,12]. E-proteins are the smallest virion structural proteins, and the five E-proteins form a pentamer transmembrane ion channel, also known as an E channel [13,14]. The S-protein comprises three S1–S2 heterodimers. The receptor binding domain (RBD) located on the head of S1 can bind to the host cellular receptor ACE2 to trigger viral infections, followed by fusion of the membrane between the viral envelope and host cell [3,4,15].

**Fig. 1:**
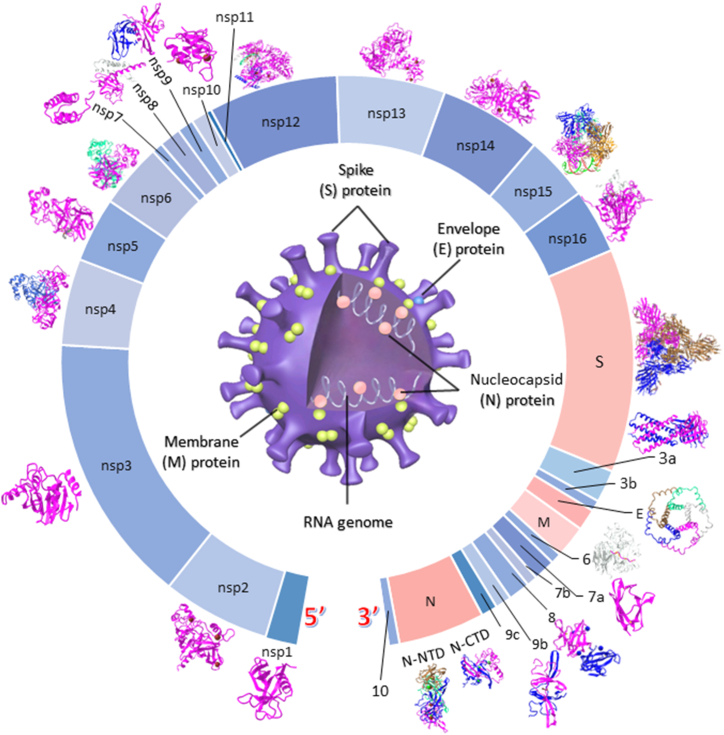
The graphic portrays the genome of SARS-CoV-2.

The genome of SARS-CoV-2 comprises several open reading frames (ORFs) separated by transcriptional regulatory sequences (TRSs) (Fig. 1). The 5′ two-thirds of the SARS-CoV-2 genome is ORF1. Genomic ORF1 comprises ORF1a and ORF1b. ORF1 can be translated into three polyproteins (PPs): PP1a, PP1b, and PP1ab. The PPs translated from ORF1 are further subjected to post-translational cleavage by two viral proteases into 16 non-structural proteins (nsp1–nsp16). These two viral proteases are papain-like proteinase (PL^pro^) and 3-chymotrypsin-like protease (3CL^pro^), also known as the main proteases (M^pro^). The first three cleavage sites of PP1a or PP1ab are cleaved by PL^pro^ to produce nsp1–nsp3, and the other post-translational cleavage of PP is achieved by 3CL^pro^ to produce nsp4–nsp16 [8,16]. nsp1 binds with type-I interferon (IFN) to affect and inhibit virus-induced IFN-β production, wherein the host immune response is inhibited to erect the viral infected cell [17]. nsp2 participates in the viral genomic replication and transcription of SARS-CoV-2 and interacts with nsp7 and nsp8 to form a replication-transcription complex (RTC). nsp3 is the largest non-structural protein (nsp) of SARS-CoV-2 with PL^pro^ activity, and is a virus-encoded protease that acts on the post-translational cleavage to produce nsp1, nsp2, and nsp3 of SARS-CoV-2 [8,18]. nsp4 is a transmembrane scaffold protein that interacts with nsp3 and nsp6 to promote the formation of double-membrane vesicles (DMVs) during viral replication [19]. nsp5 is 3CL^pro^ (or M^pro^), and serves as a post-translational cleavage site for the production of most SARS-CoV-2 nsps [20]. nsp7 and nsp8 comprise the accessory cofactors of the RNA-dependent RNA polymerase (RdRp), and nsp9 serves as a single-stranded RNA-binding protein that plays a role in viral genome reproduction [[21], [22], [23]]. nsp10 is a zinc finger protein that binds to non-specific RNA to serve as a cofactor for nsp14 and nsp16 [24,25]. nsp11 is an accessory remnant polypeptide of PP1 formed by the post-translational cleavage of only 13 amino acid residues [26]. nsp12 is an RdRp, namely, the core protein that binds to its essential cofactors nsp7 and nsp8 to form the RTC [27]. nsp13 catalyzes the unwinding of duplex oligonucleotides into single strands in a nucleotide-binding pocket (NTP)-dependent manner [28]. nsp14 is a bifunctional enzyme of SARS-CoV-2 with two active domains: the N-terminal exonuclease (ExoN) and C-terminal guanine-N7 methyltransferase (N7-MTase) domains. The ExoN of nsp14 is important for proofreading, elongating RNA, and excising mismatched bases during viral replication and transcription, and nsp10 serves as a cofactor to stimulate ExoN activity [29]. N7-MTase of nsp14 is an S-adenosylmethionine-dependent methyltransferase that catalyzes genomic RNA cap synthesis [30,31]. The C-terminal domain of nsp15 acts as a nidoviral RNA uridylate-specific endoribonuclease (NendoU) that cuts single-stranded RNA to evade viral detection by the host's defense system [22,32,33]. nsp16 of SARS-CoV-2 is a S-adenosyl-l-methionine (SAM)-dependent MTase that methylates the RNA cap at ribose 2′-O positions (2′-*O*-MTase). nsp16 binds with its cofactor nsp10 modify the 5′-end of viral RNAs to simulate the host mRNA structure, which is imperative for viral genomic replication and expression, and aids in the viral immune evasion of coronavirus [34,35]. All the viral functional proteins of SARS-CoV-2 may serve as antiviral therapeutic targets for the development of new pharmaceuticals.

## 3 The significance of quercetin against COVID-19

Quercetin (3,30,40,5,7-pentahydroxy-2-phenylchromen-4-one) is a major component of the flavonoid subclass. In particular, it is found in many common fruits and vegetables. Among vegetables, high levels of quercetin have been found in red leaf lettuce (*Lactuca sativa* L.; 10.3–30.6 mg/100 g), asparagus (*Asparagus officinalis* L.; 23.6 mg/100 g), Romaine lettuce (*Lactuca sativa* L.; 12 mg/100 g), and onions (*Allium cepa* L.; 11–41.9 mg/100 g); among fruits, apples (2.3 mg/100 g), cherries (1.2 mg/100 g), and blueberries (3.58 mg/100 g) have high contents of quercetin [36,37]. Quercetin has been found to possess multiple biological activities, including anti-allergic, anti-inflammatory, anti-oxidative, and anti-aging properties [38,39]. Notably, the potential pharmacological properties of quercetin are as follows: antiallergic, anti-diabetic, anticancer, promotion of anti-oxidant activities [[40], [41], [42]], anti-inflammatory, antimicrobial, immunoprotective effects [43], neuroprotective properties, prevention of chronic diseases [44], anti-obesity, antineoplastic, inhibition of lipid peroxidation, platelet aggregation, capillary permeability, and stimulation of mitochondrial biogenesis [[45], [46], [47]]. Similar to most other polyphenols, quercetin has a low rate of oral absorption, and its clinical use is considered to be of modest utility. Based on the low bioavailability of quercetin, innovative drug delivery strategies, such as quercetin polymeric micelles, quercetin nanoparticles, glucan-quercetin conjugates, and quercetin-loaded muco-adhesive nanoemulsions, have been suggested to increase the bioavailability of quercetin and facilitate novel therapeutic approaches [2,48]. Quercetin may be more applicable in new preparations for human healthcare after improvements in its solubility and bioavailability [49,50].

Quercetin is a nuclear factor erythroid-derived 2-like 2 (Nrf2)-interacting nutrient that reduces insulin resistance, endothelial damage, lung injury, and cytokine storms. Quercetin and some quercetin derivatives also play a role in certain mechanisms, such as the mammalian target of rapamycin (mTOR), peroxisome proliferator-activated receptor γ (PPARγ), nuclear factor kappa B (NF-κB), extracellular signal-regulated kinases (ERK), and elongation initiation factor 2α (eIF2α). Some quercetin derivatives are important in mitigating the severity of COVID-19 by acting through endoplasmic reticulum stress or the ACE-Angiotensin–II–AT1 receptor axis (AT1R) pathway [51,52], which is an Nrf2-interacting nutrient that also interacts with TRPA1 and/or TRPV1 [53]. First, Nrf2 agonists abolish the replication of SARS-CoV-2 in lung cells, and quercetin is a potent Nrf2 agonist. Second, quercetin exhibits antiviral activity against several zoonotic coronaviruses, including SARS-CoV-2, by hindering the entry of virions into host cells. Third, inflammasomes are cytosolic multi-protein complexes that generate active forms of cytokines IL-1β and IL-18. Quercetin suppresses the activation or inhibition of the nod-like receptor (NLR) family pyrin domain-containing 3 (NLRP3) inflammasomes by affecting mediators, such as thioredoxin-interacting protein, sirtuin 1, and NRF2 [44]. Inflammatory pathways are activated by inflammasome, and NF-κB and interleukin-6 signals provoke the cytokine release syndrome, which promotes ARDS in COVID-19 patients, and Quercetin may inhibit these pro-inflammatory signals [54]. Fourth, patients with COVID-19 can develop thrombosis, and quercetin alleviates coagulation abnormalities by inhibiting plasma protein disulfide isomerase [55]. Quercetin and some quercetin derivatives have multi-target binding abilities with several viral proteins of SARS-CoV-2, which play an indispensable role in facilitating viral replication (Fig. 3). Quercetin and its derivatives target the aforementioned target proteins to block viral replication and transcription, which may be used alone or in combination with other treatments to enhance therapeutic efficacy [56].

Multiple variants of SARS-CoV-2 have been found, raising questions regarding the efficacy of different lines of treatment, such as vaccines and antiviral drugs. Notably, many small molecules extracted and isolated from plants are known for their antiviral effects [57,58], whereas no antiviral drugs from plants have been approved thus far. Evaluating the safety and effectiveness of herbal medicines is difficult because of their adverse effects and herb-drug interactions [59,60]. To explore appropriate prevention/treatment measures, antibacterial, antifungal, and antiviral properties were verified in mammalian HEK293T cells [61], indicating that quercetin and some quercetin derivatives with immunomodulatory activity play a pertinent role in reducing inflammation and thwarting serious chronic diseases. This analysis indicated that although positive data were obtained, they were still insufficient to recommend quercetin and quercetin derivatives as potential immunomodulatory agents against COVID-19. Additionally, an essential enzyme in the SARS-CoV-2 replication machinery, namely, RdRp, was targeted in a virtual screening assay using a set of 1664 FDA-approved drugs, including botanical and synthetic derivatives. Notably, a set of 22 drugs with high docking scores may be candidates as inhibitors of RdRp of SARS-CoV-2 [62]. Among these candidates, quercetin and several phytocompounds have been approved by the FDA for use as anti-oxidants, cell-protective agents, general tonics, immune stimulants, and other miscellaneous systemic or topical applications.

**Fig. 2:**
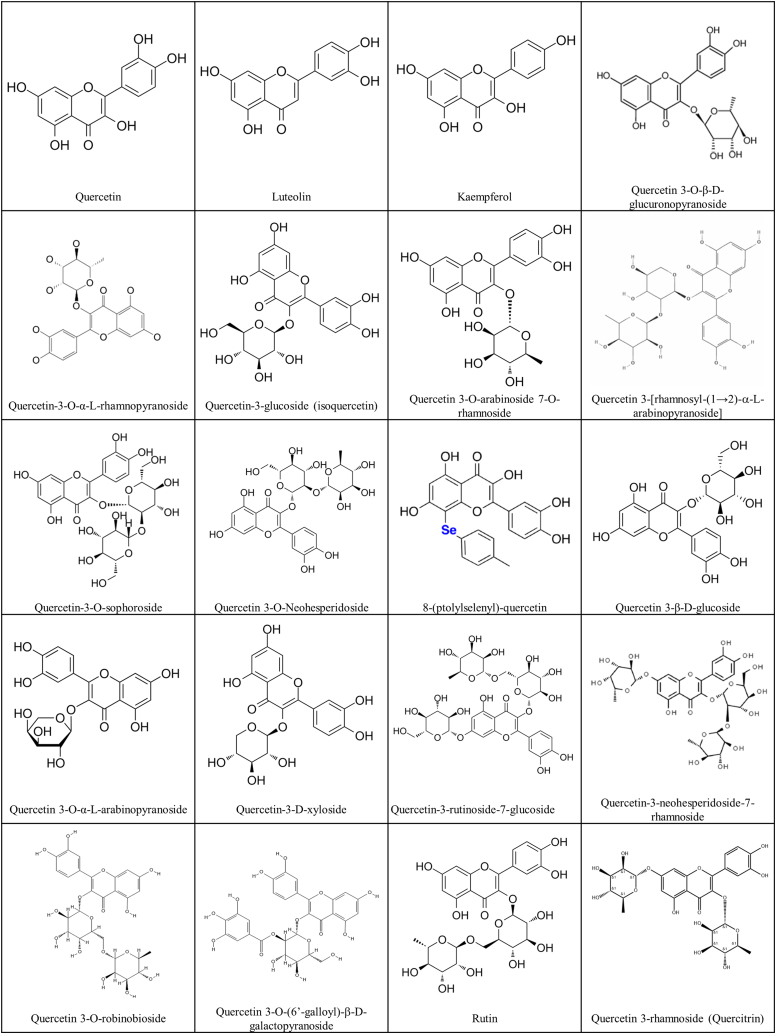
The illustration depicts the structure of quercetin and its derivatives. All share the same core structure.

The epidemiological burden of COVID-19 is a healthcare challenge worldwide as it tested the limits of medical capacity and preventive strategies and methods. Based on the identification of relevant key structure-activity relationships (SARs), analogs of quercetin and its derivatives have superior anti-inflammatory, anti-oxidative, and antiviral effects compared with the original molecules. These approaches highlight the mechanisms that govern their anti-inflammatory, anti-oxidant, and antiviral activities [63]. One strategy could be to enhance the antiviral immune response through a nutritious diet, including quercetin and quercetin derivatives isolated from natural extracts, to minimize the risk of infection (Fig. 2). Through a critical analysis of the literature, quercetin and its derivatives were found to act concurrently on inflammation and viral and oxidative stress, which may alleviate life-threatening diseases. Considering the increasing threat of COVID-19, the therapeutic efficacy of existing antiviral molecules in edible fruits should be evaluated; their potential anti-oxidant, anti-inflammatory, and immunomodulatory effects could help determine prophylactic and adjuvant therapies [64].

**Fig. 3:**
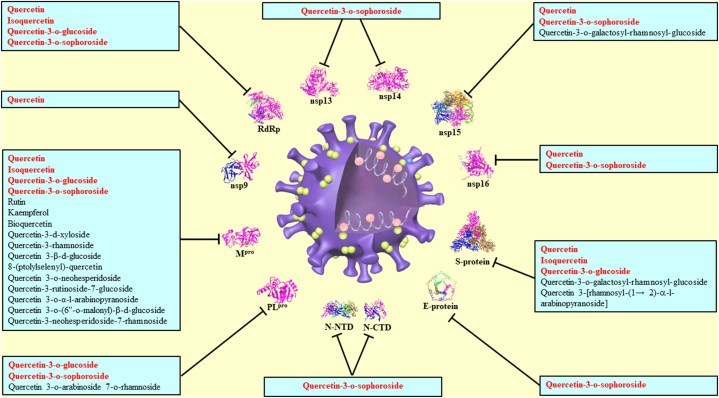
Quercetin and its derivatives docked on various domain or segment proteins of SRAS-CoV-2. Red indicates the dominated compounds. The black T bar present suppressed compounds. (For interpretation of the references to colour in this figure legend, the reader is referred to the Web version of this article.)

In this review, we collected literature and subsequently analyzed and discussed the current state of knowledge regarding the antiviral properties of quercetin and its derivatives, wherein the underlying mechanisms of action were focused on major human viruses belonging to the family Coronaviridae. We collected literature from various data sources, such as Google Scholar, Medline, Scopus, and PubMed. The purposes of molecular docking, in-silico, and virtual screening (simulation) were separated into several aspects: the option of target (multi-target), compounds (multi-ligand), and signal transduction (multi-pathways) to show evidence of crosstalk and other relevant mechanisms (Fig. 3).

## *4* *In silico* investigations of quercetin and its derivatives on COVID-19

### 4.1 *In silico* of quercetin on targets of SARS-CoV-2

Molecular docking demonstrated multiple binding sites or different affinity scores for the same ligand/target protein and various programs were used or conditions set in many studies. However, the results from *in silico* studies provide predictive information on molecular interactions between ligands and target proteins along with the real effects still needed for further validation from *in vitro, in vivo*, and *in clinical* studies. There were more than 100 studies through Jan 2024 that had investigated the effects or medicinal potential of quercetin and its derivatives on COVID-19 pandemic based on molecular docking results. Notwithstanding, the docking evidence showed quercetin interact with different viral proteins of SARS-CoV-2, which affects viral infections and replication. It also acts on several host-based proteins as early as possible to attenuate infections and symptoms for COVID-19.

The genomic expressions of ORF-1 in SARS-CoV-2 are translated into polyproteins and the cleavage into several functional proteins (non-structural proteins, nsp1-nsp16) by two major viral proteinases, 3CL^pro^ and PL^pro^ (2), and in fact 3CL^pro^ of SARS-CoV-2 is also called M^pro^. The application of multi-target approach is employed for the termination of the viral life cycle. To explore the bioactive compounds in honey, the results presented quercetin as acting and forming hydrogen bonds with Tyr54, Leu141, Ser144, His163, and Gln189 residues for active binding sites in M^pro^ [65]. For screening bioactive compounds from Ayurvedic medicinal plants, this study conformed quercetin to form a hydrogen bond within the active site of M^pro^ [56]. A molecular docking study on the bioactive constituents from *Cressa cretica* revealed quercetin forms hydrogen bonds within the binding sites of M^pro^ at Thr24, Thr26, Asn28, His41, Asn119, Asn142, and Gln189 residues [66]. Another study showed the quercetin can form hydrogen bond interactions with the activation site and dimerization site of M^pro^ at Glu288, Asp289, and glu290 residues, as well as form a π-Alkyl interaction with regulatory site at Leu286 [67]. The results demonstrated that quercetin interacts with M^pro^ [[68], [69], [70], [71]], major predicted binding sites at three amino acid residues Asp48, Met49, and Asp187 of M^pro^ [69]. The *in silico* predicted study revealed quercetin binds with M^pro^ at Tyr237, Asn238, Gly275, Met276, and Gly278 residues [72]. For example, (quercetin/M^pro^) interactions at amino acid residues at Asn142, Gly143, Ser144, Leu141, Cys145, His41. and Thr26 [73]. In another study, the substrate binding pocket of M^pro^ binds by rutin, quercetin, and kaempferol at the catalytic residues of His41 and Cys145 with high affinity (10^−5^-10^−6^ M) [74]. The results from the above studies showed that even with similar docking scores or binding energy, but the interacting residues for quercetin on M^pro^ only few matched from various experiments. Additionally, A molecular docking studies on the investigation of inhibitor for SARS-CoV-2 showed that quercetin also can form five hydrogen bond interactions with PL^pro^ at AspP77, Arg83, Thr75, and Asn157 amino acid residues [75]. Such massively divergent results further indicated that for *in silico* outcomes, in particular, for M^pro^ were significantly affected by target protein model choice, molecular docking program used, operational and conditional setting, bias or training of researchers, and all *in silico* results need further validation from either direct evidence from a binding assay or indirect approach by *in vitro* or *in vivo* studies for further confirmation. These will be discussed in another section of this paper.

Another study illustrated that quercetin interacts with RBD of S-protein and also forms hydrogen bonds with the s2 domain of S-protein at Ala1056, Pro1075, Gly1059, His1058, Ser730, Met731, Lys733, and Leu861 residues [76]. Quercetin is a major bioactive component for several Ayurveda natural products, molecular docking results showed quercetin acts on S-protein by forming hydrogen bonds with Gln314, Ser316, Asn317, Asn764, Arg765, and Thr768 residues with a binding affinity of −14.48 kcal/mol [77]. Quercetin forms three conventional hydrogen bonds with amino acid residues at Gly496, Asn501, and Tyr505 in S-protein (PDB: 6LZG) of SARS-CoV-2, the pi-pi interaction at Tyr453, and pi donor hydrogen bond at Arg403. In addition, quercetin generates van der Waals interactions with Gln506, Phe497, Tyr495, Gln493, Lys417, Glu406, and Leu155 [67].

Nsp13 is a functional helicase of SARS-CoV-2 that provides the 5′-triphosphatase and RNA helicase activities [78]. *In silico* simulations show quercetin simultaneously docks with two major functional pockets of SARS-CoV-2 nsp13: the NTP and 5′-RNA-binding sites, which interrupts the viral RNA unwinding and ATPase activity; and the *in vitro* enzymatic activity test confirmed the potential of quercetin as candidate as for a powerful inhibitor of nsp13 [79]. SARS-CoV-2 nsp15, an endoribonuclease, is a crucial viral protein for the mechanistic action of viral infection, replication, and transcription; and it constrains production of the host, IFN-β [80]. The results presented show that quercetin forms several hydrogen bonds with nsp15 at four amino acid residues Lys290, His235, His250, and Glu340 [73].

*In silico* evidence showed that quercetin had a high binding affinity with nsp16 by forming hydrogen bonds or π-anion interactions with four amino acid residues at Asp130, Gly73, Leu100, and Asp99 [81]. In general, intracellular RNA will be degraded by RNase of eukaryotic cells for SARS-CoV-2 survival, while the modification of cap methylation at the 5′end of SARS-CoV-2 RNA genome plays a vital role in its prevention as it is degraded by the host intracellular protection mechanisms. The methylation on the 5′-cap of SARS-CoV-2 RNA genome assumes key roles on the viral replication and immune evasion, which is catalyzed by nsp14 (guanine-N7 methyltransferase) and nsp16 (2′-*O*-ribose methyltransferase) [30]. The RNA 5′ end cap modification of SARS-CoV-2 were carried by the nsp10/nsp16 RNA cap 2′-*O*-Methyltransferase [25]. Additionally, quercetin acts as the anti-cap methyltransferase inhibitor on the enzymatic active site of nsp16 at Tyr47, Gly71, Asp114, Phe149, and Asp130 residues via molecular docking [82] (Table 1).

**Table 1:** Quercetin and its derivatives docking with targets of host and SARS-CoV-2.

Ligand	Host/virus	Target	PDB ID	binding site	Reference
**Quercetin**	Host	ACE2	1R42	Lys745, Tyr613, His493, Asp609	[165]
				Tyr385, Phe390, Arg393, Asp350	[77]
			1R4L	Arg273, Phe274, His345, Pro346, Thr347, Ala348, Thr371, His374, Glu375, His378, Glu406, Phe504, His505, Tyr515, Arg518	[97]
		STAT1	2KA6	Met28, Asn93	[166]
		TNF	5M2J	Phe144, Leu142, Ala22, Gly24	
		GRP78	5E84	Glu427, Gly430, Thr458, Lys460	[167]
		IL-6	1ALU	Arg30, Arg 179, Leu33, Leu178, Arg182	[126]
			n.a.	Glu127, Gln130, Glu137, Arg141	[106]
		IL-1α	n.a.	Asn92, Glu149, Lys150, Tyr153, Tyr175	
		TNF-α	n.a.	Leu113, Gln201	
		TMPRSS2	UniProt O15393	Gly383, Gly385, Asn433, Asp435, Asn398, Gly259, Ala400	[99]
		TMPRSS2	7MEQ	Lys390, Gln438, Ser436, Cys465, Trp461 Cys437, Gly464, Cys465	[100]
		FOS	1FOS	Ser154, Arg155, Ser278, Arg279, Asn156	[107]
		p38/MAPK	5XYY	Lys53, Thr106, His107, Met109, Asp112, Asp168	[104]
		JNK2	3E7O	Glu109, Met111, Gln117	
	SARS-CoV-2	M^pro^ (nsp5)	3IWM	Tyr237, Asn238, Gly275, Met276, Gly278	[72]
			6LU7	Leu141, Gly143, Ser144, Cys145, His163, Met165, Arg188	[88]
			2A5I	Tyr54, Glu55, Asp187	[168]
			4XFQ	Ala26, Cys144, Ile140, Asn141, His162	[169]
			5R84	Arg298	[68]
			5RFS	Thr190, His41, Met49, His164, Met165, Glu166, Pro168, Gln192	[170]
			6LU7	His164, His41, Cys44, Met49, Tyr54, Cys85, Cys145, Met165, Gly174, Thr175, Phe181, Phe185, Val186, Asp187, Arg188, Gln189, Gln192	[56]
				Thr24, Thr26, Asn28, His41, Asn119, Asn142, Gln189	[66]
				Glu166, Gln189, Gln192	[132]
				Arg131, Lys137, Thr199, Tyr239, Leu286, Asp289	[120]
				Leu141, Gly143, Ser144, Glu166	[167]
				Thr26, His41, Asn142, Leu141, Gly143, Ser144, Cys145	[73]
				Tyr54, Leu141, Ser144, His163, Gln189	[65]
				Tyr54, Glu166, Gln192, Thr190, Asp187, His41, Met165, Leu167, Arg188, Gln189, Pro168	[171]
				Thr26	[113]
				Gly143, Glu166, Arg188, Glu189	[172]
			6YB7	Thr199, Glu288, Glu290, A sp289, Leu286	[67]
			6Y2E, 6Y2F	Glu166, Leu141, His41, Cys145	[118]
			6Y84	Gln127, Cys128, Lys137, Asp289, Glu290	[145]
			6W9C	Asp157	[173]
				Gln30	[168]
			2GTB	Thr111, Asp153	
			6LU7, 5C3N	Leu50, Gln189	[91]
		S-protein	6LZG	Arg454, Arg457, Lys458, Asp467, Ser469, Glu471	[88]
			6LZG	Gly496, Asn501, Tyr505, Tyr453, Arg403, Gln506, Phe497, Tyr495, Gln493, Lys417, Glu406, Leu155	[67]
			6M17	Ser349, Leu441, Asp442, Asn448, Asn450, Arg509	[167]
			6VXX	Gln314, Ser316, Thr768, Asn317, Asn764, Arg765	[77]
				Asp88, Asp198, Ile233, Ile235	[168]
			6VW1	Thr445, Ile446	[113]
			6VYB	Ser730, Met731, Lys733, Leu861, Ala1056, Pro1057, His1058, Gly1059, Val860, Pro863, Asp867, Met730, Ile870,	[76]
			6VSB	Thr549, Asp745, Asn978, Arg1000	[75]
		S-protein (Closed)	6VXX	Tyr741	
		S-protein (Open)	6YVB	Tr549, Asn978, Gly744, Arg1000, Thr573, Met740, Tyr741	
		nsp16	6YZ1	Gly73, Leu100, Asp130, Glu71, Ala72, Ser74, Asp75, Ser98, Asp99, Leu100, Asp114, Cys115, Met131, Tyr132, Asp133, Phe149	[81]
			6WJT, 6WKQ	Tyr47, Gly71, Asp114, Phe149, Asp130	[82]
		RdRp (nsp12)	6M71	Glu350, Asn628, Val315, Arg349, Pro461, Pro677	[120]
			6NUS	Glu350, Pro323, Asn628, Val675	[75]
			7BTF, 6M71, 6NUR	Tyr619, Cys622, Asp623, Asp761, Ser841	[168]
			6W9Q	Leu45, Thr109	
		nsp9	6W4B	Pro58, Thr68	
		nsp15	6VWW	Lys290, His235, His250, Glu340	[73]
			6W01	Leu347, Lys346, Asn279, Cys292	[174]
		PL^pro^ (nsp3)	3E9S	Lys158, Asp165, Thr169	[168]
		S-protein/ACE2	6M0J	Ala348, Gly352, Asp382, Phe390, Arg393, Asn394, His401	[120]
Rutin	Host	ACE2	1R4L	Asn149, Met270, His345, Lys363, Thr445, Tyr127, Leu144, Glu145, Glu150, Met152, Ala153, Asp269, Trp271, Phe274, Thr276, Cys344, Pro346, Met360, Cys361, Asp367, Asp368, Thr371, Leu503, Phe504	[97]
		IRF3	1QWT	Leu196, Gly199, Glu201, Pro223, Glu224, Arg276, Gly278	[102]
	SARS-CoV-2	M^pro^ (nsp5)	5RFS	Thr25, Thr24, Gly143, His164, Asn142, Thr26, Cys145, Arg188, Met165, Asp187, Met49, Gln189, Glu166	[170]
			6LU7	Asn142, Thr190, His41, Met49, Glu166, Asp187, Arg188, Gln189	[70]
				His164, Gln192, Asp187, Glu166, Leu141, Pro168, Ala191, Cys44, Pro168	[175]
			6Y2E, 6Y2F	His41, Cys145, Glu166, Leu141	[118]
quercetin-3-*O*-glucoside	SARS-CoV-2	PL^pro^ (nsp3)	4OVZ	His74, Arg83, Tyr155, Asn157, His176	[75]
		M^pro^ (nsp5)	6LU7	Leu141, His163, Met 165	
		RdRp (nsp12)	6NUS	Ala125, His133	
		S-protein	6VSB	Phe970, Arg995, Thr998	
		S-protein (Closed)	6VXX	Glu988	
		S-protein (Open)	6YVB	Thr998, Arg995, Asp994	
Quercetin 3-*O*-(6″-galloyl)-β-d-galactopyranoside	Host	IRF3	1QWT	Glu201, Thr219, Ile220, Ser221, Glu293, Ile395, Asp392, Ser398	[102]
Quercetin-3-*O*-galactosyl-rhamnosyl-glucoside	SARS-CoV-2	RBD of S-protein	6M0J	Glu406, Gln493, Gly496, Gln498, Gly502, Tyr505	[110]
		Nsp15	6W01	Gln245, Val339, Glu340, Trp333, Met219, Ala232, Gly230, Gly247, Gly248	
Quercetin-3-rutinoside-7-glucoside	SARS-CoV-2	M^pro^ (nsp5)	5RFS	Thr24, Thr25, Thr26, Ser46, Asn142, His164, Glu166, Gly143, Leu27, Thr45, Met165, His41, Met49, Arg188, Leu167, Pro168	[106]
Bioquercetin (Quercetin 3-*O*-robinobioside)	SARS-CoV-2	M^pro^ (nsp5)	6LU7, 5C3N	Pro168, Gln189	[91]
Quercetin-3-D-xyloside	SARS-CoV-2	M^pro^ (nsp5)	6LU7	His41, Leu141, Asn142, Ser144, His163, Glu166, Thr190, Met165, Pro168, Asp187, Arg188, Gln189	[70]
Quercetin 3-*O*-α-l-arabinopyranoside	SARS-CoV-2			Tyr53, His163, Glu166, Asp187, His41, Met49, Phe140, Leu141, Arg188, Gln189	
Quercetin 3-*O*-(6″-*O*-malonyl)-β-D-glucoside	SARS-CoV-2			Leu141, Ser144, Glu166, His41, Met49, Asn142, Cys145, Met165, Asp187, Arg188, Gln189	
Quercetin 3-β-D-glucoside	SARS-CoV-2			Met49, Pro52, Asn142, Glu166, Leu167, Thr169, Gln189, Met49, Pro52	[111]
8-(ptolylselenyl)-quercetin	SARS-CoV-2	M^pro^ (nsp5)	6Y2E, 6Y2F	Gln189, His41, Cys145,	[117]
quercetin-3-rhamnoside	SARS-CoV-2	M^pro^ (nsp5)	6Y2E	Lys5, Ala7, Gln127, Lys137, Glu290	[151]
Isoquercetin	SARS-CoV-2	M^pro^ (nsp5)	5RFS	Glu166, Asn146, His41, Gly143, Leu27, Thr25, Cys44, Val42, Met49, Cys145, Met165, Arg188, Gln189, Thr190, Pro168, Leu167, Leu141, Ser144, His163, Phe140	[106]
		S-protein	6VSB	Phe347, Val350, Asp442, Phe497, Arg509, Arg346, Ala348, Val401, Ser349, Gln493, Tyr495, Tyr351, Ser494, Gln498, Pro499, Ser443	
		RdRp (nsp12)	6M71	Trp617, Asp618, Tyr619, Asp760, Lys798, Pro620, Lys621, Cys622, Cys799, Trp800, Gly616, Ala762, Asp761, Glu811, Ser814, Ser759	
Quercetin 3-*O*-Neohesperidoside	SARS-CoV-2	M^pro^ (nsp5)	6LU7	His41, Gly143, Cys145, Gln189, Thr190	[85]
quercetin 3-*O*-arabinoside 7-*O*-rhamnoside	SARS-CoV-2	PL^pro^ (nsp3)	4M0W	Lys27, Gln41, Arg42, Arg72, Arg74, Asn157, Lys158, Glu162, His176	[87]
quercetin-3-neohesperidoside-7-rhamnoside	SARS-CoV-2	M^pro^ (nsp5)	6LU7	Asn133, Thr169, Ala194, Asp197, Thr199, Asn238, Leu287	
quercetin 3-[rhamnosyl-(1- > 2)-alpha-l-arabinopyranoside]	SARS-CoV-2	S-protein	6LZG	Ala348, Tyr385, Asn394, Glu398, Arg514	
Quercetin-3-*O*-sophoroside	SARS-CoV-2	M^pro^ (nsp5)	6LU7	Asn119, Asn142, Gly143, Cys145, His164, Met165, Glu166, Asp187, Gln189,	[144]
		RdRp with RNA	7BV2	Asp452, Arg555, Thr556, Lys621, Cys622, Asp623, Ser682,	
		RdRp without RNA	7BV2	Tyr619, Asp760, Ser759, Arg553, Arg555, Thr556, Val557, Ser682, Ser681, Thr680, Arg624, Thr687, Cys622, Lys621, Asp623	
		Nsp14 ExoN	5C8S	Asp90, Val91, Glu92, Asn104, Pro141, Gln145, Ala178, Phe190, Glu191, Leu235, Asn252, His268, Asp273,	
		Nsp14 N7-MTase	5C8S	Cys309, Arg310, Gln313, Gly333, Asn334, Pro335, Lys336, Ile338, Lys339, Trp385, Cys387, Phe401	
		Nsp15	6WLC	His234, Gly246, Gly247, His249, Ser293, Trp332, Glu339, Thr340, Tyr342, Pro343, Lys344	
		Nsp16 (2′-*O*-MTase)	6WVN	Asn29, Tyr30, Lys46, Gly71, Ala72, Gly73, Ser74, Asp75, Asp99, Leu100, Asp130, Met131, Tyr132, Pro134, Lys170, Glu203	
		PL^pro^ (nsp3)	6WUU	Lys157, Gly160, Glu161, Leu162, Leu199, Met206, Tyr207, Met208, Gly209, Gln221, Ile222, Tyr268, Gln269	
		E protein	Epro.pdb	Phe23, Phe26, Leu27, Leu28, Val29, Thr30, Leu31, Ala32, Thr35, Cys43, Ile46, Val47, Leu51	
		Nsp13 ADP site	6JYT	Glu261, Gly285, Thr286, Gly287, Ser289, His290, Lys320, Lys322, Tyr324, Thr440, Arg442	
		Nsp13 NCB site	6JYT	Glu143, Arg178, Asn179, Val181, Thr228, His230, Cys309, Ser310, His311, Arg339, Asn361, Pro408, Arg409, Thr410,	
		N protein NCB site	6YI3	Arg41, Pro42, Leu45, Asn48, Thr49, Ala50, Ser51, Ala90, Thr91, Tyr109, Pro117, Pro151,	
Luteolin	Host	IL-6	1ALU	Ser176, Arg182, Leu178, Arg179	[126]
		STAT1	2KA6	Asp24	[102]
		IL-2	5LQB	Lys52, Thr57	
		TNF	5M2J	Asp45	
Kaempferol	Host	NF-κB	1IKN	Gly31, Alh192, Asp185	[102]
		IκB-α	1NFI	Gln212, Gly206, Arg245	
		IL-6	1ALU	Arg179, Arg182, Leu178, Arg30	[126]
	SARS-CoV-2	M^pro^	6LU7	Try54, Glu166, Met165, Met49, Cys145, His41	

*n.a as none available in cited References.

*Epro.pdb provided by COVID-19 Docking Server (https://ncov.schanglab.org.cn/).

*Protein amino acid residues interact by hydrogen bonds with ligands labeled in blue based on the indications of cited References.

### 4.2 *In silico* of quercetin derivatives on targets of SARS-CoV-2

A molecular docking study on the interaction with the active site of M^pro^ to screen anti-COVID-19 potential of the phytocompounds from the *Acacia pennata* had results that showed several flavonoids with a quercetin-similar chemical backbone as C6–C3–C6 structure act on the active site of M^pro^ [83]. Based on evidence from *in silico* simulations, many natural putative derivatives of quercetin and kaempferol both act on RdRp and M^pro^, promising rutinoside flavonoids affecting SARS-CoV-2 replication [84]. The interaction between Quercetin 3-*O*-neohesperidoside and M^pro^ binding pockets is from the forming hydrogen bonds at Gly143, Cys145, Gln189, Thr190, and His41; and forming π-π interaction at His41 residues. The interaction between Quercetin 3-Rhamnoside and M^pro^ by forming hydrogen bonds at same amino acid residues as Quercetin 3-*O*-neohesperidoside [85]. The molecular docking simulation showed quercetin and its derivatives interacts with SARS-CoV-2 M^pro^ active sites but binds on the regulatory site, dimerization site, or other positions to indicate quercetin and its derivatives may inhibit or affect M^pro^ activity as the competitive or non-competitive inhibitor [67,86], but this needs additional experiments to verify it.

The results provide a high binding affinity between S-protein/quercetin 3-(rhamnosyl-(1 → 2)-α-l-arabinopyranoside], PL^pro^/Quercetin 3-*O*-arabinoside 7-*O*-rhamnoside, and 3CL^pro^/Quercetin-3-neohesperidoside-7-rhamnoside [87]. Quercetin 3-*O*-arabinoside 7-*O*-rhamnoside forms 9 hydrogen bonds with PL^pro^ via 9 amino acid residues at Lys27, Gln41, Arg42, Arg72, Arg74, Asn157, Lys158, Glu162, and His176. Quercetin-3-neohesperidoside-7-rhamnoside forms 10 hydrogen bonds with 3CL^pro^ via 7 amino acid residues Asn133, Thr169, Ala194, Asp197, Thr199, Asn238. and Leu287. Quercetin 3-[rhamnosyl-(1 → 2)-α-l-arabinopyranoside] forms 7 hydrogen bonds with S-protein via 5 amino acid residues Ala348, Tyr385, Asn394, Glu398, and Arg514 [87]. Quercetin-3-Orutinoside and quercetin-7-*O*-glucoside-3-*O*-rutinoside are metabolic products of quercetin glycoside that have the highest binding affinity with the M^pro^ of SARS-CoV-2 [84]. The chemical structure of myricetin has one more hydroxy group than quercetin that forms the interaction with M^pro^ at same amino acid resides at Leu141, Gly143, Ser144, Cys145, His163, and Met165 as quercetin [88]. Quercetin-3-glucoside is a monoglycosylated quercetin derivative with higher water solubility than quercetin, also called isoquercetin [89]. Isoquercerin forms double hydrogen bonds with two amino acid residues at Csy145 and Asn142 in the active site of M^pro^ to provide strong inhibition [90]. Another *in silico* study showed isoquercetin interacts with multiple viral proteins of SARS-CoV-2 to form hydrogen bonds with M^pro^ at Glu166, Asn146, and His41 residues, forms hydrogen bonds with S-protein at Phe347, Val350, Asp442, and Phe497 residues, and forms hydrogen bonds with RdRp at Trp617, Asp618, Tyr619, Asp760, and Lys798 residues [86]. Quercetin 3-*O*-robinobioside kaempferol, and quercetin are phytochemicals that could be isolated from *Boerhavia diffusa* with similar quercetin chemical backbones that may show the inhibiting effect on the M^pro^ by molecular docking simulation [91].

Quercetin acts on viral proteins to affect COVID-19 infection and *in silico* results have revealed that several quercetin derivatives act on the viral proteins of SARS-CoV-2 [92]. Quercetin can biosynthesis from kaempferol or de-glycosylation from rutin which has the same chemical backbone structure with only one different R-group (Fig. 2). The natural polyphenols luteolin and myricetin have a similar chemical backbone to quercetin, *in silico* docking simulation showed that the two compounds similarly interacted with S-protein RBD of SARS-CoV-2 at the amino acid residues Arg454, Arg457, Lys458, Asp467, and Glu471 [88] (Table 1).

Recently, the Omicron variant of SARS-CoV-2 quickly worldwide, molecular docking calculation showed quercetin 3-*O*-β-d-glucuronopyranoside and quercetin-3-*O*-glucuronide 6″-*O*-methyl ester may have a strong inhibition on the RBD of Omicron variant [93]. Moreover, the binding affinity of quercetin and its metabolic derivatives toward furin enzymes or S-protein of SARS-CoV-2 may be affected by the micro-environmental pH value. Increasing the pH value may cause deprotonation on quercetin to decrease the number of hydrogen bond formations and increase the length of hydrogen bounds to drop its inhibitory activity on viral proteins [94].

### 4.3 *In silico* of quercetin and its derivatives on targets of host cells

Quercetin forms binding interaction with viral non-structural proteins of SARS-CoV-2 as well as has a high binding affinity with COVID-19 reacted proteins of host, such as ACE2, ABL1, and TMPRSS2 [92,95]. ACE2 and NRP1 as the host viral receptors for the S-protein of SARS-CoV-2 binding triggers the viral infection and enter host cells, so one promising strategy to prevent SARS-CoV-2 infection is interrupted or blocked the interaction between S-protein and ACE2/NRP1 [92]. The first molecular docking study about quercetin acting on ACE2 and S-protein of SARS-CoV-2 was reported [96] and it provided evidence for quercetin having a high binding affinity with both targets: host ACE2 and viral S-protein by forming hydrogen bonds with ACE2 at Arg273, Asp269, Asn149, and Tyr127 residues. Further that quercetin forms hydrogen bonds within the binding pocket of RBD of S-protein at Lys462, Glu465, Arg466, Asp146, and Ile123 residues [96]. I*n silico* evidences that quercetin affords a high binding affinity with S-protein by forming hydrogen bonds or π-π interactions with four amino acid residues Asn501, Tyr505, Gly496, and Tyr453 within the ACE2 binding packet of RBD [67], and several quercetin derivatives can simultaneously act on all three proteins of ACE2, NRP1, and S-protein to prevent protein-protein interactions between S-protein and ACE2/NRP1 receptors [92]. Interestingly, MLN-4760 is an ACE2 inhibitor that acts on the extracellular metallopeptidase domain of ACE2, the molecular docking results showed (rutin and quercetin) interacted with amino acid residues of ACE2 are similar to MLN-4760 (the natural inhibitor of ACE2) while have a higher binding affinity than MLN-4760 [97]. Quercetin acts on ACE2 by forming hydrogen bond interactions at Asp350, Tyr385, Phe390, and Arg393 residues with the binding affinity as −7.86 kcal/mol [77]. In addition to ACE2, the host TMPRSS2 also served an important function on the initial stage of SARS-Co-V-2 infection [3]. The coronavirus infects by first binding to ACE2 (a serine protease acting as the receptor), while another serine protease is necessary for priming the viral S-protein required for entering the cells. The TMPRSS2 of human cells play a significant role in proteolytic cleavage of SARS-Cov-2 S-protein and subsequent priming to the receptor ACE2. The catalytic domain with serine protease function is located at C-terminus of TMPRSS2, and it cleaves viral S-protein to facilitate the membrane fusion between SARS-CoV-2 and the host cells [98]. Based on the results of molecular docking calculation, the first report showed the inhibiting effect of quercetin on the TMPRSS2 because quercetin forms strong hydrogen bonds with TMPRSS2 at Gly383, Gly385, Asn398, Asn433, and Asp435 residues; weak carbon-hydrogens interaction at Gly259 and Asn398 residues; and π-alkyl bond at Ala400 [99]. Another molecular docking study indicated quercetin forms strong hydrogen bond interactions with the core functional amino acid residues of TMPRSS2 at Lys390, Gln438, Ser436, and Cys465 residues, and forms the π-π interactions at Cys437 and Trp461 that near the catalytic site of TMPRSS2 [100].

### 4.4 *In silico* of quercetin and its derivatives on inflammation and its targets of host cells

Host cells infected by SARS-CoV-2 may cause oxidative stresses, cell injury, cytokine storm, serious inflammations, and organ functional damages are the major etiological factors of COVID-19 pathogenesis. Quercetin forms interactions with different viral proteins of SARS-CoV-2 to affect the virus replication and acts on several host-based proteins to attenuate infected symptoms of COVID-19 pandemic. SARS-CoV-2 infection caused the activation of NF-κB signaling pathway, which leads to upregulating several inflammatory factors synthesis and release [101]. ORF8 of SARS-CoV-2 is a viral protein with 121 amino acids that forms the ORF8 dimer or interact with the host protein IRF3 or MHC-I to form ORF8-IRF3 protein complex or ORF8-MHC-1 complex [102,103]. The ORF8 dimer, ORF8-IRF3 complex and ORF8-MHC-1 complex may hijack the induction of host immune system, ORF8-IRF3 complex may inhibit NF-κB signal pathway to decrease IFN production and attenuate the downstream immune response [102]. Rutin and Quercetin 3-*O*-(6′-galloyl)-β-d-galactopyranoside can dock on the interface residues between OFR8 and IRF3 to disturb the formation of ORF8-IRF3 complex, and that may help virus scavenging by host immune responses [102]. It postulates that the promotion of host immunity against viral invasions.

Quercetin, kaempferol, and luteolin are the major bioactive components identified from Yindan Jiedu granules (YDJDG), which have an inhibiting effect on NF-κB signaling pathway [75]. The results found quercetin binds with NF-κB by hydrogen bonds at four residues: Gly31, Arg33, Thr191, and Ala192; and with IκB-α by hydrogen bonds at Lys177, Arg245, and Asp208 residues. The luteolin binds with NF-κB by hydrogen bonds at Gly31, Asp185, and Lys218 residues, and with IκB-α hydrogen bond at Gln 212 residues. The kaempferol binds with NF-κB by hydrogen bonds at three residues: Gly31, Alh192, and Asp185; and with IκB-α by hydrogen bonds at Gln212, Gly206, and Arg245 residues [75].

Quercetin inhibits the protein expressions of NLRP3 inflammasome pathway to decrease pro-inflammatory cytokine expressions as curcumin does [78], and the docking results confirmed quercetin forms hydrogen bonds within the ATP binding sites of p38/MAPK at Lys53, Thr106, His107, Met109, Asp112, and Asp168 residues; and binding with the ATP binding pocket of JNK2 at Glu109, Met111. and Gln117 residues [104]. The activation and expression of intracellular STAT3, PI3K, and MAPK are key targets for anti-inflammation. Molecular docking showed quercetin and luteolin interacts with STAT3, PIK3R1, and MAPK1 [105].

### 4.5 Immune response of quercetin and its derivatives on targets of host cells via *in silico*

The hyper-activation of the immune system causes an acute severe systemic inflammatory response called cytokine release syndrome (CRS). No effective prophylactic or post-exposure treatments are available, although some anti-inflammatory compounds are currently in clinical trials. The cytokine storm is one of the main causes of mortality by severe SARS-CoV-2 infection. The increase expressions of IL-1β, IL-6 and TNF-α levels were found in the pro-inflammatory status of SARS-CoV-2 infection, and quercetin interacts with those three pro-inflammatory cytokines to attenuate the downstream inflammatory pathways. The molecular docking calculation showed the strong interaction between quercetin and IL-1β by formed 6 hydrogen bonds with 5 amino acid residues at Asn92, Glu149, Lys150, Tyr153, and Tyr175. The quercetin interacts with TNF-α by two hydrogen bonds at Leu113 and Gln201. Quercetin forms a stable interaction with IL-6 by hydrogen bonds at the amino acid residues Glu127, Gln130, Glu137, and Arg141 [106].

One study reported that patients infected by SARS-CoV-2 may increase CCL2, CXCL8, FOS, IFN-β, IL-1A, IL-1B, and SERPINE1 gene expressions, suggesting that it participates in cytokines, inflammation, and oxidative stress pathways. Molecular docking results shows that quercetin acts on the proteins CCL2, CXCL8, FOS, IL-1α, IL-1β, and SERPINE1; rutin acts on CXCL8 and IL-1β; apigenin acts on FOS and SERPINE1; and baicalein acts on FOS [107]. Quercetin acts on the anti-inflammatory heme oxygenase-1 (HO-1) pathway to inhibit the expressions of TNF-α, IL-1β, IL-6, and NO production in the serum to attenuate inflammation induced by infection [108,109]. In the HO-1 pathway, quercetin did not affect the expression of kelch-like ECH-associated protein-1(Keap1), but *in silico* result showed quercetin binds on the Nrf2 binding site of Keap1 and leads to the expressions of downstream anti-oxidative genes [110]. Additionally, several quercetin and its derivatives (quercetin, kaempferol, luteolin, myricetin, and apigenin) were isolated from *Platycladus orientalis* form hydrogen bonds within the active site of xanthine oxidase to inhibit the enzyme activity and reactive oxygen species (ROS) production [111]. A total of 214 active ingredients and 339 target genes of Shufeng Jiedu capsules (SFJDC) were collected. Of note (quercetin, kaempferol, luteolin) and 10 hub target genes (TP53, AKT1, ANXA1, CTNNB1, NCOA1, EGFR, PRKCA, NCOA2, RELA, and FOS) were identified based on network analysis. The hub target genes are mostly augmented in pathways including MAPK signaling pathway, PI3K-Akt signaling pathway, and cAMP signaling pathway, which could be the underlying pharmacological mechanisms of SFJDC for treating COVID-19. Moreover, the key compounds had a high binding activity with three typical target proteins including ACE2, 2OFZ, and 1SSK [112].

### 4.6 *In vitro* and *In vivo* validation

To verify the results of *in silico* simulation, some of the studies performed further *in vitro* and *in vivo* tests. The cytotoxicity of quercetin on Vero E6 cells with 50 % cytotoxicity concentration (CC_50_) value as 301.5 μM. The anti-COVID-19 activity with 50 % inhibitory concentration (IC_50_) value as 18.2 μM. The selectivity indexes (SI=CC_50_/IC_50_) of quercetin are about 17 and it had multiple molecular docking interactions with several SARS-CoV-2 viral proteins that indicate quercetin as a potential pharmaceutical candidate for SARS-CoV-2 treatment [113]. The SARS-CoV-2 infection was initiated by the viral S-protein binding with host membrane ACE2 receptor, ligands bind on the ACE2 may affect or interrupt the COVID-19 virus infection. The *in vitro* studies that were performed by surface plasmon resonance (SPR) technology showed the equilibrium dissociation constant (Kd) of quercetin binding with ACE2 is 5.92 μM with the IC_50_ of quercetin on recombinant human ACE2 was 4.48 μM [114], and several quercetin related glycosides also possessed the potential to be inhibitors of ACE2 [115]. The nsp13 of SARS-CoV-2 is a helicase for viral replication, quercetin and several flavonoids had a high binding affinity at the 5’ RNA binding site, the inhibiting effect of 30 μM quercetin completes blocking of the ATPase activity of nsp13. Quercetin, myricetin, and kaempferol inhibited SARS-CoV-2 nps13 through an unwinding activity at nanomolar concentrations but rare effects on viral replication [79].

The genomic expressions of ORF-1 in SARS-CoV-2 are translated into polyproteins and cleaved into functional proteins (non-structural proteins, nsp1-nsp16) by two major viral proteinases, M^pro^ and PL^pro^ [116]. The inhibitions on the M^pro^ activities may inhibit the viral replication and infection, and an *in vitro* result demonstrate quercetin acts on the 3CL^pro^ as the inhibitor with IC_50_ value range from 6.79 to 23.4 μM in different reports, and EC_50_ is 4.88 μM [69,71]. *In vitro* cell culture results showed natural quercetin inhibited SARS-CoV-2 replication in Vero cells at a relatively high concentration with the IC_50_ as 192 μM, and the quercetin modified into seleno-quercetin derivative can significantly improve the inhibiting effect on SARS-CoV-2 replication, and the best IC_50_ is 8 μM [118]. This shows that using quercetin as the structural backbone to modify and develop new bioactive compounds may provide more powerful pharmaceutical candidates for anti-SARS-CoV-2 treatments. Quercetin, and kaempferol are the major flavonoids of *Hippophae rhamnoides*. The result of an SPR assay showed the binding affinity of quercetin to human ACE2 protein with 5.92 μM of Kd, and the binding affinity of isorhamnetin was higher than quercetin with 2.51 μM of Kd. By using the SARS-CoV-2 spike pseudotyped virus assay, treated with 50 μM isorhamnetin inhibits 47.7 % viral entrance but treated by the same dose of quercetin had no effect [114].

Rutin is the quercetin with disaccharide retinose, also called quercetin-3-*O*-rutinose. Rutin is more water soluble and has more bioavailability than quercetin, which forms the interactions with M^pro^ at similar binding residues. *In vitro* M^pro^ activity shows that form an inhibiting test that is estimated to show that IC_50_ of rutin at about 32 μM [118]; and mixed rutin with l-arginine improves with more water solubility and its inhibiting effect on M^pro^ to decrease the Ki and IC_50_ values [119]. Another *in vitro* study used HCoV-229E to simulate SARS-CoV-2, the results showed the inhibitory effects of quercetin, isoquercetin, and rutin on M^pro^ activity of SARS-CoV-2 with 6.79 μM, 4.03 μM and 0.125 μM of IC_50_, respectively. The substitute test from the evaluation of the inhibiting effect on HCoV-229E replication in Huh-7 cells conformed with the antiviral effects of quercetin, isoquercetin, and rutin [71]. Another *in vitro* study also validated the ligands-protein interactions of SARS-CoV-2 by using fluorescence quenching methods and showed that the IC_50_ of quercetin on RdRp, M^pro^ and S-protein about 82 nM, 62 nM and 52 nM, respectively, as well as catechin had similar anti-COVID-19 potential. Its inhibiting efficacy on RdRp, M^pro^ and S-protein with 71 nM, 93 nM and 65 nM of IC_50_, respectively [120]. The nsp15 is uridylate specific endoribonuclease of SARS-CoV-2 that cleaved the viral own negative-sense RNA to evade the innate immune response [121]. *In vitro* results showed epigallocatechin gallate (EGCG) and baicalin inhibits nsp15 with 1.67 μM and 3.56 μM of IC_50_, respectively [122]. Further, another *in vitro* study tested the anti-COVID-19 effects of different combinations that mixed quercetin with different natural compounds to indicate the combination by resveratrol, broccoli extract, curcumin, quercetin, naringenin, baicalin, theaflavin, vitamin C, and N-acetylcysteine had the highest anti-SARS-CoV-2 potential. *In vitro* treatments with this combination (10 μg/mL) inhibited 90 % S-protein binding to ACE2, and 53 % RdRp activity to inhibit SARS-CoV-2 infection and replication [123]. Another *in vitro* study showed the inhibitory effect (IC_50_) of quercetin about 23.8 μM on M^pro^ and 8.7 μM on PL^pro^ of SARS-CoV-2, respectively [124]. Additionally, the *in vitro* validation showed quercetin had a dose-dependent inhibiting effect on M^pro^, used the non-linear regression to establish inhibition constant (K_i_^app^) of quercetin on M^pro^ catalytic activity is 21 μM, and intrinsic inhibition constant (K_i_) is 9.6 μM [125]. Direct binding evidence by an SPR assay showed that the equilibrium dissociation constants (Kd) value of quercetin on ACE2 was 0.67 mM; quercetin forms hydrogen bonds within the binding pocket of RBD of S-protein at Lys462, Glu465, Arg466, Asn146, and Ile123 residues with 2.21 μM of Kd [96] (Table 2).

**Table 2:** The anti-SARS-CoV-2 targets and effects of Quercetin and its derivatives.

Target		Quercetin/Quercetin derivatives	IC_50_ (μM)	CC_50_ (μM)	Method	Reference
SARS-CoV-2	M^pro^	Quercetin	6.79		Measured by SARS-CoV-2 Assay Kit (BPS bioscience)	[71]
		Kaempferol	6.83			
		Isoquercitrin	4.03			
		Rutin	0.125			
	M^pro^	Quercetin	23.4		Measured by FRET	[69]
	M^pro^	Quercetin	21		Measured by FRET	[117]
	M^pro^	Rutin	32		Measured by FRET	[118]
	M^pro^	Quercetin	62 nM		Measured by spectrofluorimetric assay	[120]
	RdRp	Quercetin	82 nM			
	nsp13	Quercetin	10.2	>100	Measured by nsp13 unwinding associated activity	[79]
	nsp15	Quercetin	13.79		Measured endoribonuclease activity by carboxyfluorescein fluorophore	[122]
	S-protein	Quercetin	52 nM		Measured by spectrofluorimetric assay	[120]
HOST	rh-ACE2	Quercetin (2.5 min)	4.48		Measured rhACE2 activity by fluorogenic substrate Mca-APK(Dnp)	[115]
		Quercetin (10.5 min)	29.5			
	ACE2	Quercetin (KD)	5.92		Measured by SPR assay	[114]
Vero E6 cells	SARS-CoV-2 replication	Quercetin	18.2	301	Measured by MTT assay	[113]

FRET: fluorescence resonance energy transfer; SPR: surface plasmon resonance; MTT: 3-[4,5-dimethylthiazol-2-yl]-2,5 diphenyl tetrazolium bromide.

There are several marketed herbal products developed in China or other countries that claimed antiviral activities with some suggested as having potential against COVID-19, but only few have been validated. *In vitro* results showed that when pretreated with herbal extracts of Jingyin granules (HEJG) there was an inhibiting effect on different drug-metabolizing enzymes (such as cytochrome P450 enzymes-CYPs) with the dose-dependent manners to affect pharmacokinetics of clinical medicines, which further indicated that cotreatment with HEJG and anti-viral drugs may increase the effective dose and prolong effective time for clinical drugs (such as lopinavir). Within HEJG, kaempferol and quercetin are major bioactive constituents of HEJG with inhibiting effects on the CYP3A [126]. Lopinavir is a general clinical medicine used for COVID-19 treatment. Rats pretreated with HEJG (3 g/kg) showed an inhibition of the activities of CPYs to influence the pharmacokinetic parameters of lopinavir to increase the maximum concentration in serum, prolong the half-life of antiviral drugs about 1.91 times, and extend the retention time [127]. Isoquercetin has higher water soluble, and the pharmacokinetic results showed oral administration with isoquercetin had over a 4-fold bioavailability when compared with quercetin *in vivo* [89]. Taken a deep insight into looking above sections of findings from *in silico*, preclinical tests and clinical trial, quercetin and its derivatives docked with various domain or segment proteins of SRAS-CoV-2 exert the suppression of inflammation and cytokine storm by coronavirus infected to combat COVID pandemic (Fig. 3).

## 5 Pharmaceutical potentials of quercetin and its derivatives in clinical trial of COVID-19 pandemic

Based on the ClinicalTrials. gov website (https://clinicaltrials.gov/), in 15 clinical trial enrollments, quercetin supplementation was used as a COVID-19 treatment. We also found other reports of six studies in PubMed (https://pubmed.ncbi.nlm.nih.gov/). In all clinical trials, quercetin was used as a supplement combined with standard care (SC) for symptomatic and antiviral treatment of COVID-19 patients (Table 3, Table 4). Outpatients with mild SARS-CoV-2 infections were treated with quercetin for two weeks (600 and 400 mg/daily of quercetin in the first and second weeks, respectively) in combination with SC, which significantly shortened the period of viral clearance, alleviated symptom occurrence, and attenuated the levels of lactate dehydrogenase (LDH) and ferritin in serum [128]. In the early stages of COVID-19, quercetin supplementation resulted in marked improvements in the ratio of hospitalized patients, decreased the oxygen needs of patients, and shortened the days of hospitalization [129]. Patients with severe SARS-CoV-2 infection were treated with 1000 mg/day of quercetin in combination with clinical antiviral drugs (such as remdesivir and favipiravir), which significantly decreased the levels of q-C-reactive protein (q-CRP), LDH, and alkaline phosphatase (ALP) in the serum and reduced the period of hospitalization [130].

**Table 3:** Clinical trial of quercetin on COVID-19 treatment from PubMed and ClinicalTrial.gov.

Formulation/Design	Phase	Regimen (Oral)	Patients (n)	Masking	Age (y)	Placebo (n)	Country	Authors or Institute	Serial number/(Other ID or DOI)
Quercetin Phytosome	3	400 mg/daily	152/200	none	>18	–	Pakistan	Ikram Din Ujjan	NCT04578158/(LUMHS/REC/894)
Quercetin Prophylaxis	–	500 mg/daily	447	none	>18	–	Turkey	Hasan Onal	NCT04377789/(KSSEAH--0058)
Quercetin Treatment		1000 mg/daily							
Quercetin Phytosome	–	600 mg/daily 1st week, 400 mg/daily 2^ed^ week	100/142	none	>18	–	Pakistan	King Edward Medical University	NCT04861298/(192/RC/KEMU)
Quercetin Treatment	4	500 mg/daily	60	none	>18	–	Saudi Arabia	Hiba Hamadelnil elsaeed	NCT04468139/(20–95 M)
Zinc Treatment		50 mg/daily							
Bromelain Treatment		500 mg/daily							
Vitamin C Treatment		1000 mg/daily							
Quercetin/curcumin combination treatment	–	–	50	none	>18	–	Pakistan	King Edward Medical University	NCT05130671/(785/RC/KEMU)
Quercetin Treatment	–	500 mg, twice daily	80	double	>18	40	Italy	Mariangela Rondanelli	NCT05037240/(1222/01022021)
Curcumin, Quercetin and Vitamin D3 combination treatment	–	curcumin168 mg, quercetin 260 mg, vitamin D3 360 IU	50	none	>18	–	Pakistan	Liaquat University Hospital	NCT04603690/(LUMHS/REC/173)
Quercetix	1	one tablet twice daily (Acetyl-l-carnitine 500 mg, quercetin 200 mg, vitamin B 12 33 mg, Vitamin E 10 mg)	80	double	>18	40	Tunisia	Hôpital Universitaire Sahloul	NCT04851821/(Quercetix)
Isoquercetin	2	1000 mg/daily	200	triple	>18	100	France	Pascal Chanez	NCT04622865/(AB20001)/(2020-001635-27)
Masitinib,		3 mg/kg/day for 4 day, follow 4.5 mg/kg/day							
QUERCOV	1	one tablet twice daily	200	triple	13–59	100	Tunisia	Riadh Boukef	NCT04853199/(QUERCOV)
Extract Psidii guava	3	2 Capsule Extract Psidii guava, three times daily	90	double	>18	45	Indonesia	Fredia Heppy	NCT04810728/(PSIDII0520_COV19)
Isoquercetin	2	Isoquercetin 1000 mg on day 1, then 500 mg	150	single	>18	75	USA	Institut de Recherches Cliniques de Montreal	NCT04536090/(IRCM-IQC-001)
NASAFYTOL	–	1008 mg, 4 capsules twice a days	51	none	>18	25	Belgium	Tilman S.A.	NCT04844658/(CHOPIN)
Quercetin	–	1000 mg/daily	60/72	none	32–79	30	Iran	Shohan M	IRCT20200419047128N2 [130],
Yindan Jiedu granules	–	12 or 24 g, 3 times daily	262/270	none	18–86	–	China	Fang Y	China, No. Z20200012000
Quercetin Phytosome® (lecithin-based delivery form, quercetin 200 mg in 500 mg tablet)	–	3 tablets/daily for 1st week, 2 tablets/daily for 2^ed^ week	42/50	none	>18	21	Pakistan	Di Pierro F	192/RC/KEMU [128]
	3	2 tablets/daily	152	none	>18	76	Pakistan	Di Pierro F	NCT04578158 [129]
Shufeng Jiedu	–	2.08 g, 3 times daily	76	none	>18	33	China	Xia L	YJ-92 2020-S015–02 [132]
multi-component OTC	–	25 mg zinc; 10 drops of Quina™; 400 mg quercetin; 1000 mg vitamin C; 1000 IU (25 mg) vitamin D3; 400 IU Vitamin E; and 500 mg l-lysine	113	none	30–59	60	USA	Margolin L	[133]

“-“ as no mention.

**Table 4:** Clinical trial results of quercetin on COVID-19 treatment from PubMed and ClinicalTrial.gov.

Formulation (daily, Oral)	Patient (n)	Age (y)	Country	Authors	Results (intervened 7th day)	Serial number
Quercetin 200 mg/tab, 2 tab/daily	152/200	18∼80	Pakistan	Di Pierro et al.	Decrease hospitalization days, non-invasive oxygen therapy, ICU, and death	NCT04578158 [128]
Quercetin 200 mg/tab, 2 tab/daily	100/108	47.6 ± 15.7	Pakistan	Di Pierro et al.	Decrease RT-PCR viral positive; Speedy clinical recovery; Decrease LDH level	192/RC/KEMU; NCT04861298 [136]
Quercetin 200 mg/tab, 3 tab/daily	42	49.3 ± 16.5	Pakistan	Di Pierro et al.	Decrease RT-PCR viral positive; Decrease LDH, Ferritin, CRP, and D-dimer levels	192/RC/KEMU; NCT04861298 [129]
Quercetin 500 mg/tab, 2 tab/daily	60	35∼75	Iran	Shohan et al.	Decrease RT-PCR viral positive and post-intervention discharge; Decrease the symptoms of fever, weakness and lethargy; Decrease ALP, q-CRP, and LDH levels	IRCT20200419047128N2 [130]
Curcumin (168 mg) Quercetin (260 mg) Vitamin D3 (9 μg)	50	46–67	Belgium	Gérain et al.	Decrease hospitalization, ICU, and COVID-19 WHO ordinal outcome score; Increase discharged participants	B4062020000305 [137]
Curcumin (168 mg) Quercetin (260 mg), Vitamin D3 (9 μg)	56/66	32∼52	Pakistan	Khan et al.	Decrease RT-PCR viral positive, symptom of cough; Decrease CRP level	785/RC/KEMU; NCT05130671 [138]
Curcumin (168 mg) Quercetin (260 mg) Vitamin D3 (9 μg)	50/64	30∼50	Pakistan	Ujjan et al.	Decrease RT-PCR viral positive, symptoms of myalgia, asthenia and dysgeusia; Decrease hs-CRP, D-dimer, LDH, Ferritin levels	LUMHS/REC/137; NCT04603690 [139]

The anti-COVID-19 efficacy of several traditional Chinese or herbal medicine patent prescriptions (TCMPPs or THMPPs) was determined within three years (2020–2022); however, they were applied or passed a clinical trial in only a few cases. Quercetin is the major bioactive component of TCMPPs and THMPPs. Yindan Jiedu granules (YDJDG) are TCMPPs originating from China, and clinical trials have shown that treatment with YDJGG (12 or 24 g, three times per day) suppressed the progression of COVID-19 based on the 1:1-ratio propensity score matching (PSM). Notably, quercetin is an effective component of YDJDG and modulates the NFκB pathway to attenuate inflammation [131]. Shufeng Jiedu capsules (SFJDC) are another type of THMPPs developed in China. In COVID-19 patients, antiviral therapy combined with SFJDC supplementation (2.08 g, three times daily) significantly alleviated coughing and fatigue symptoms, improved rehabilitation, and increased asymptomatic periods during the entire viral infection period compared with antiviral therapy alone [132].

In one clinical trial report, a multi-component over-the-counter (OTC) drug (comprising 10 drops of Quina™, 400 mg of quercetin, 25 mg of zinc, 1000 mg of vitamin C, 1000 IU (25 mg) of vitamin D3, 400 IU of vitamin E, and 500 mg of l-lysine) was used to treat early-stage COVID-19 patients. When supplied with OTC drugs, infected patients showed an asymptomatic status and a decreased ratio to become symptomatic [133]. Other studies on the effects and mechanisms of vitamin D_3_, quercetin, and estradiol on anti-inflammation, anti-oxidation, and antiviral infection indicated that their use in a tripartite combination is a potential mitigation agent of COVID-19 [134,135]. Quercetin possesses strong anti-inflammatory, anti-oxidant, immunomodulatory, and antiviral characteristics, which are considered to indicate a high safety profile, as determined by preclinical studies on animals and humans. Recently, quercetin was used as an adjuvant therapy in patients with COVID-19, and the results showed an improvement in clinical symptoms and a reduction in the length of hospitalization [128] (Di Pierro et al., 2021a). To date, only three different quercetin formulations have been investigated in clinical trials and published, with seven reports in PubMed, to evaluate the therapeutic and prophylactic potential of quercetin against SARS-CoV-2 infection (Table 4). Based on these reports, the administration of quercetin in SARS-CoV-2-infected patients can enhance recovery and decrease the acute and serious infective symptoms [[128], [129], [130],[136], [137], [138], [139]]. Most clinical reports have showed that in comparison with those in the control group, quercetin intervention could attenuate the levels of inflammatory biomarkers in patient serum on the 7th day after intervention [129,130,138,139].

## 6 To accelerate the prospective of in-silico findings

Currently, to rapidly track the urgent demand for treatments regardless of the COVID-19 pandemic or chronic diseases and syndromes, namely, diabetes, metabolic syndrome, and hypertension, a new bioinformatics approach has garnered research interest to determine the therapeutic effect in drug discovery and development, and the underlying mechanism of small molecules in signaling pathways. In several studies, network pharmacology was combined with molecular docking to investigate the potential compounds of traditional Chinese medicine (TCM) formulas for SARS-CoV-2 therapies. Many in-silico studies have shown that quercetin acts on the target proteins of SARS-CoV-2 and the concomitant pathway of COVID-19 pathogenesis [135,140,141]. Quercetin, luteolin, and kaempferol not only bind and inhibit M^pro^, but can also dock on nine therapeutic targets, namely, IL-6, tumor necrosis factor (TNF), microtubule associated protein kinase 1 (MAPK1), CASP3, CXCL8, IL-10, IL-1β, MAPK8, and VEGFA [126]. Quercetin, luteolin, and kaempferol are the major bioactive flavonoids in Lianhuaqingwen capsule (LHQW-C), indicating that the above mixture is a TCM formula used for COVID-19 treatment. Kyoto encyclopedia of genes and genomes (KEGG) enrichment analysis indicated that the targets of LHQW-C highly enhanced several immune response-related and inflammation-related pathways, including the TNF signaling pathway, NF-κB signaling pathway, IL-17 signaling pathway, and Th17 cell differentiation. Moreover, kaempferol, quercetin, and luteolin showed high binding affinities for 3CL^pro^ of SARS-CoV-2. One study revealed that some anti-inflammatory constituents of LHQW-C moderate the inflammatory response in severely ill patients with COVID-19 [126].

Additionally, an online COVID-19 docking server (http://ncov.schanglab.org.cn) was developed to apply small molecular ligands interacting with relative proteins of SARS-CoV-2 [142]. This docking server was used to screen the bioactive components from Mexican herbal medicines, and the simulation results showed that quercetin acts on several viral nsps and is one of the top three natural compounds with potential against SARS-CoV-2 [143]. Second, quercetin-3-*O*-sophoroside has a high binding affinity for several SARS-CoV-2 proteins, including E-protein, N-protein, nsp14, nsp15, nsp16, PL^pro^, M^pro^, and RdRp [144].

Notably, numerous studies have investigated the docking of M^pro^ with quercetin, which has different amino acid residues at each binding site. Notably, SAR can be used to explain and identify potentially important residues. All the above studies showed that quercetin develops other interactions with M^pro^ in addition to the protease active sites, and may act as a mechanism of the allosteric inhibition of M^pro^ [145]. Certain amino acids in M^pro^ play a major role in binding, as determined by many studies, whereas other studies found that allosteric regulation is likely to exist. Additionally, the in-vitro validation assay was confirmed using amino acid mutations. Moreover, illustrating the direct binding or interaction of quercetin with target molecules requires further studies with target identification methods, such as surface plasmon resonance (SPR), isothermal titration calorimetry (ITC), microscale thermophoresis (MST), drug affinity responsive target stability (DARTS), stability of proteins from rates of oxidation (SPROX), and cellular thermal shift assay (CETSA), which are applicable to natural products, as well as indirect evidence through biological and pharmacological evaluations (*in vitro* and *in vivo*) [146]. Quercetin, rutin, and isoquercetin are the major bioactive natural compounds found in Toujie Quwen Granules, and have high binding scores for IL-6, ACE2, and S-protein. Further evidence based on SPR indicates that quercetin and isoquercetin bind to the RBD of the S-protein to interrupt the attachment and binding of the S-protein to the host ACE2, whereas rutin binds to ACE2 to decrease and dwindle the SARS-CoV-2 infection [147]. The S-protein of coronavirus activates the ERK-MAPK pathway to affect ACE2 expression, thereby increasing viral infection [148]. Once SARS-CoV-2 binds to ACE2, it causes viral replication, oxidative stress, and an immune response, which subsequently mediates the p38/MAPK signaling pathway and induces the upregulation of AP-1 through ERK-pJNK to overexpress ACE2 to increase the binding of coronavirus. Moreover, the production of reactive oxygen species (ROS), inflammation, and cytokine storms resulted in the activation of JAK/stat1, inflammatory cytokines (IL, IFN, and TNF), NF-κB, and NLRP3-translocated regulation of pro-inflammatory and IFN-stimulated genes.

## 7 The possible mechanisms of quercetin when targeting SARS-CoV-2 infection

**Fig. 4:**
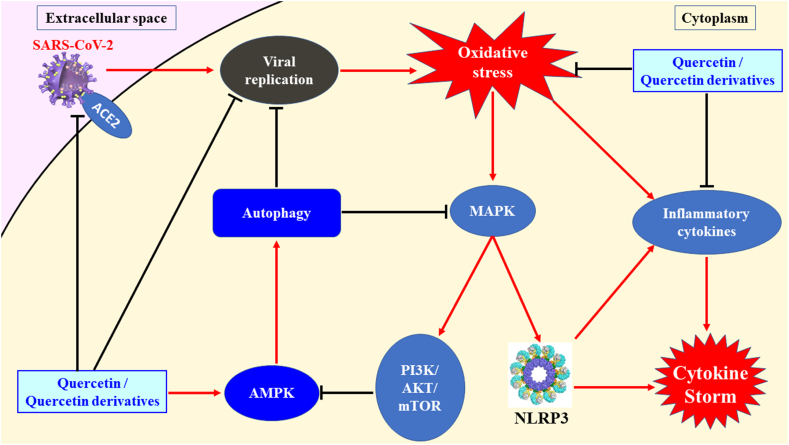
The mediation of quercetin and its derivatives on (a) the inflammation and cytokine storm, (b). The expressions of inflammatory and interferon-stimulated genes and (c) the oxidative stress induced by SARS-CoV-2 infection.

Quercetin and its derivatives act on multiple pathways, including extracellular and cytoplasmic pathways, to abrogate SARS-CoV-2 infection (Fig. 4). Based on the above literature, the major anti-COVID-19 mechanisms of quercetin and its derivatives may involve three main routes for modulating and inhibiting SARS-CoV-2 infection. First, quercetin and its derivatives can interrupt the viral-host protein interaction by interrupting and preventing viral S-protein anchoring on the host ACE2/TMPRSS2, which may block membrane fusion between the virus and host cell and prevent viral entry into the host cell. Second, quercetin and its derivatives are predicted to have multiple binding characteristics to interact with several SARS-CoV-2 proteins, especially by binding to the active sides of viral proteins, which can inhibit viral replication and attenuate host intracellular oxidative stress by viral infection. Third, quercetin and its derivatives act on several host signaling pathways to attenuate oxidative stress, inhibit the synthesis of inflammatory cytokines, and activate autophagy. Quercetin and some of its derivatives can induce autophagy inducers [149,150]. Treatment with quercetin or its derivatives can activate the AMP-activated protein kinase (AMPK) pathway to promote autophagy in infected cells, which can help host cells remove viral proteins, harmful proteins, or impaired organelles. In other studies, autophagy was shown to act on the P38/MAPK pathway to downregulate the PI3K/AKT/mTOR and NLRP3 inflammasome pathways and decrease cytokine synthesis, which may attenuate the risk of developing a cytokine storm after SARS-CoV-2 infection.

## 8 Quercetin and its derivative as an adjuvant synergistic with other drugs for COVID-19 treatment

Additionally, there is a prospect for the development of formulations using different permutations and combinations of these phytomolecules to harness their synergistic potential. In another in-silico study, the anti-COVID-19 potential of several clinical anti-human immunodeficiency virus (HIV) drugs and phytoflavonoids was screened. The results showed that synergistic treatment with phyto-flavonoids and anti-HIV drugs could increase the docking score of anti-HIV drugs for SARS-CoV-2 M^pro^. This study also indicated that synergistic treatment with quercetin-3-rhamnoside and darunavir had the highest docking score for SARS-CoV-2 M^pro^, suggesting that this combination could be used as an effective approach against SARS-CoV-2 [151]. As expected, the high incidence of post-COVID-19 symptoms was ascribed to the long-term complications of COVID-19; this simultaneously provided a complex view that emphasized multidisciplinary care and the prophylactic and therapeutic potential of such treatments. The most advantageous flavonoid, quercetin, showed significant therapeutic and preventive effects for the mitigation and shortening of the period concomitant with post-COVID-19 syndrome [151,152]. Increasing evidence has revealed that many patients develop a chronic condition characterized by fatigue, neuropsychiatric symptoms, various neurological disorders, such as headache or myalgia, and more severe symptoms, such as stroke, psychosis (brain fog), and anosmia, termed long-COVID. There is an urgent need to identify possible interventions to mitigate the crossing of the blood-brain barrier (BBB) by the S-protein, which results in perivascular inflammation. Notably, the use of small natural molecules, especially luteolin and quercetin, to cross the BBB abrogates this detrimental effect [[153], [154], [155]].

Multisystem inflammatory syndrome (MIS) is a post-viral immunological or hyperinflammatory complication of SARS-CoV-2 infection in children (MIS-C) [156], adults (MIS-A) [157], or after vaccination (MIS-V). MIS-C is commonly observed in older children and is characterized by multi-organ dysfunction involving cardiac, renal, respiratory, hematologic, and acute gastrointestinal problems; neurological symptoms; rash or bilateral non-purulent conjunctivitis; hypotension; or shock. Its main manifestations include abdominal pain, diarrhea, fever, myocarditis, and coronary artery aneurysms [158]. The average age of children with MIS-C (56.8 % of male) was 9 years. Fever (100 %), gastrointestinal (GI) (82 %), and abdominal pain (68 %) were the decisive symptoms in the diagnosis of MIS-C. Shock and/or hypotension is common in patients with MIS-C. Cardiac symptoms (66 %) are dominant over respiratory (39 %) and neurological (28 %) symptoms [159]. However, pediatric COVID-19 is still a mild disease, affecting only 8 % of children. The pathogenesis of SARS-CoV-2 infection in children is comparable with that in adults, which indicates the impaired activation of IFN-α and IFN regulator 3 and decreased cell response, causing impaired viral defense; however, the clinical course is mild, and all children recover from the infection without major complications or sequelae. Furthermore, the clinical manifestations of MIS-C resemble certain pediatric rheumatologic diseases, such as Kawasaki disease (mucocutaneous lymph node syndrome), which distress the small-medium vessels [160]. Most patients with MIS-C have increased levels of at least four inflammatory markers (C-reactive protein, neutrophil count, ferritin, procalcitonin, fibrinogen, interleukin-6, and triglycerides) [161]. Critically, MIS-C, a hyper-inflammatory syndrome associated with SARS-CoV-2 infection, shares clinical features with toxic shock syndrome (TSS), which is triggered by bacterial superantigens (SAgs). Superantigen specificity for different Vβ chains results in Vβ skewing, wherein T cells with specific Vβ chains and varied antigen specificity are overrepresented in the T cell receptor (TCR) repertoire. Notably, in-silico modeling revealed that polyacidic residues in the Vβ chain encoded by TRBV11-2 (Vβ21.3) strongly interact with the SAg-like motif of the SARS-CoV-2 S-glycoprotein, suggesting that the unprocessed S-protein of SARS-CoV-2 may directly modulate TRBV11-2 expansion [162].

The pathology of COVID-19 is still characterized by a cytokine storm, such as an increased inflammatory response involving IL-6, TNF-α, and monocyte chemoattractant proteins that lead to endothelial inflammation, microvascular thrombosis, and multiple organ failure. An acute cytokine storm in severe COVID-19 results in multi-organ damage owing to vascular hyperpermeability, edema, and hypercoagulation. The consequences of SARS-CoV-2 infection include long-COVID (i.e., long-term) or post-COVID (i.e., short-term) syndromes and MIS-C [163,164]. In particular, COVID syndrome may be characterized by long-term tissue damage (e.g., lung, brain, and heart) and pathological inflammation (e.g., viral persistence, immune dysregulation, and autoimmunity) associated with increased levels of specific biomarkers (e.g., CRP, D-dimer, and lymphocyte count) [163]. Increasing evidence has indicated persistent and/or delayed complications from symptom onset in acute COVID-19. The pathophysiology and organ-specific sequelae of post-acute viral syndromes have been described in survivors of other infectious coronavirus epidemics [164]. The sequelae of coronavirus may be attributed to the manifestations of post viral syndrome, especially post- and long-COVID.

Based on the anti-inflammatory, immune-modulated, and cardiovascular protective effects of quercetin and its derivatives, the search of phytochemicals, namely, quercetin and its derivatives, that can bind to the S-protein of SARS-CoV-2 to block the interaction with TRBV11-2 (Vβ21.3) and reduce the symptoms or occurrence of MIS-C will be ongoing. Herbal compounds (mostly polyphenols and flavonoids, namely, luteolin) are known to possess a broad spectrum of pharmacological properties, such as anti-oxidant, anti-inflammatory, and antiviral activities, suggesting their role as a micronutrient in the prevention of the inflammatory cascade to alleviate manifestations of post-COVID, long-COVID, and MIS-C.

Unfortunately, according to the global statistics of population, the percentage of vaccination is over 63 % (1), whereas the low cumulative mortality is only approximately 1 %. Compared with the rapid decline of the SARS outbreak in the past decade, the declining tendency of COVID-19 pandemic is devastating, and COVID-19 may become infectious and flu-like in the future. In contrast, the high mutation rate of SARS-CoV-2 may give rise to new immune escape variants for vaccinated or infected people, which can increase the contagious risk. Notably, this multi-targeting potential of quercetin and its derivatives on several proteins of SARS-CoV-2 can potentially help combat COVID-19 and long-COVID.

## 9 Conclusion and future prospective

We provided a comprehensive review of the in-silico, in-vitro, in-vivo, and clinical literature examining the anti-SARS-CoV-2 effectiveness of quercetin and its derivatives. The evidence from in-silico simulations showed the potential multi-targeting effect of quercetin and its derivatives on several proteins of SARS-CoV-2 and host cells, some of which have been determined to combat COVID-19 through in-vitro, in-vivo*,* and clinical settings. From May 2023, the WHO and most countries announced the end of the emergency phase of the COVID-19 pandemic, and that most people have received several vaccinations; however, the variants of SARS-CoV-2 still spread worldwide and continue to infect several million people every year (https://covid19.who.int/). Considering the cost of global actions to fight the COVID-19 pandemic, the COVID-19 pandemic never ceased but became integrated into our everyday lives, thereby indicating the necessity and urgency of developing treatment strategies that are cheap and easy to obtain. Recent anti-SARS-CoV-2 studies have demonstrated the anti-inflammatory, anti-oxidative, neuroprotective, and cardiovascular protective effects of quercetin and its derivatives, suggesting their preventive and therapeutic potential against the ongoing COVID-19 pandemic and COVID-19-associated diseases and complications, such as post-COVID, long-COVID, and MIS-C.

The authors declare that they have no known competing financial interests or personal relationships that could have appeared to influence the work reported in this review.

## Ethics approval and consent to participate

Not applicable.

## Human and animal rights

Not applicable.

## Consent for publication

Not applicable.

## Availability of data and materials

Not applicable.

## Funding

The work was supported by Xiamen Medical College Research Grant (k2020-07; k2019-01; k2019-03) and Education and Scientific Research Project for Middle-aged and Young Teacher in Fujian Province, Department Education, Fujian (JAT200735 for Xiao-hui Zheng).

## CRediT authorship contribution statement

**Wan-Yi Ho:** Formal analysis. **Zi-han Shen:** Validation, Supervision, Software, Resources, Project administration, Methodology. **Yijing Chen:** Visualization, Supervision, Formal analysis. **Ting-Hsu Chen:** Software, Resources, Project administration. **XiaoLin Lu:** Data curation. **Yaw-Syan Fu:** Writing – review & editing, Writing – original draft, Visualization, Data curation.

## Declaration of competing interest

The authors declare that they have no known competing financial interests or personal relationships that could have appeared to influence the work reported in this paper.

## Figures and Tables

**figs1:**
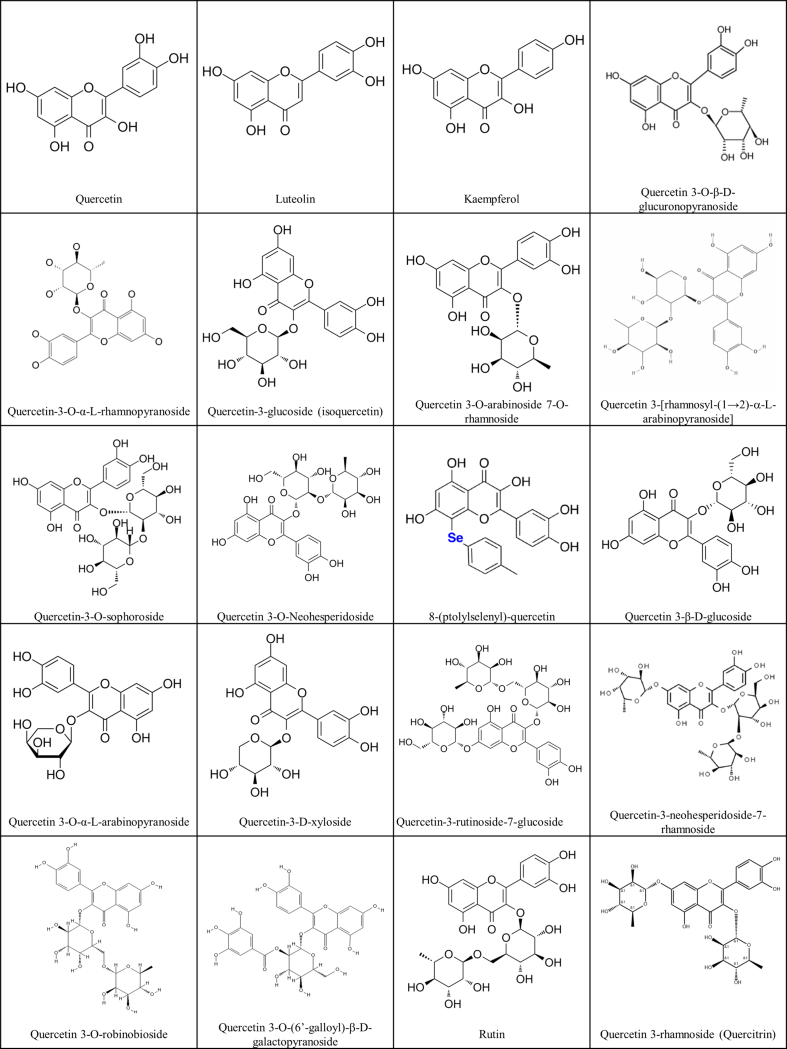


## Abbreviation

SARS-CoV-2: Severe acute respiratory syndrome coronavirus 2
COVID-19: coronavirus infectious disease 2019
ACE2: angiotensin-converting enzyme 2
TMPRSS2: transmembrane serine protease 2
RBD: receptor binding domain
ORFs: open reading frames
TRSs: transcription regulatory sequences
PPs: polyproteins
PLpro: papain-like proteinase
3CLpro: 3-chymotrypsin-like protease
Mpro: main protease
IFN: type-I interferon
RTC: replication-transcription complex
DMVs: double-membrane vesicles
RTC: replication-transcription complex
RdRp: RNA-dependent RNA polymerase
ExoN: N-terminal exonuclease domain
N7-Mtase: C-terminal guanine-N7 methyltransferase
NendoU: nidoviral RNA uridylate-specific endoribonuclease
SAM: S-adenosyl-l-methionine
2′-*O*-MTase: MTase to methylate the RNA cap at ribose 2′-O positions
Nrf2: nuclear factors erythroid-derived 2-like
mTOR: mammalian target of rapamycin
PPARγ: peroxisome proliferator-activated receptor γ
NF-κB: nuclear factor kappa B
ERK: extracellular signal-regulated kinases
eIF2α: elongation initiation factor 2α
AT1R: ACE-Angiotensin–II–AT1 receptor
SARs: relevant key structure activity relationships
YDJDG: Yindan Jiedu granules
CRS: cytokine release syndrome
HO-1: heme oxygenase-1
Keap1: kelch-like ECH-associated protein-1
ROS: reactive oxygen species
CC50: 50 % cytotoxicity concentration
IC50: 50 % inhibitory concentration
EGCG: epigallocatechin gallate
HEJG: herbal extracts of Jingyin granules
SC: standard care
LDH: lactate dehydrogenase
PSM: propensity score matching
SFJDC: Shufeng Jiedu capsules
OTC: over-the-counter
LHQW-C: Lianhuaqingwen capsule
nsps: non-structure proteins
SAR: surface activity relationship
ITC: isothermal titration calorimetry
MST: microscale thermophoresis
DARTS: drug affinity responsive target stability
SPROX: stability of proteins from rates of oxidation
CETSA: cellular thermal shift assay
MIS: multisystem inflammatory syndrome
GI: gastrointestinal
TSS: toxic shock syndrome
TCR: T cell receptor
